# Low-Carbohydrate Diet and Metabolic Syndrome Risk in Korean Adults: A Korea National Health and Nutrition Examination Survey Study

**DOI:** 10.3390/nu18010178

**Published:** 2026-01-05

**Authors:** Vasuki Rajaguru, Jeoungmi Kim, Durga Datta Chapagain, Tae Hyun Kim, Sang Gyu Lee, Whiejong M. Han

**Affiliations:** 1 Department of Healthcare Management, Graduate School of Public Health, Yonsei University, Seoul 03722, Republic of Korea; vasuki@yuhs.ac; 2 Department of Nursing Science, Kaya University, Gimhae 50830, Republic of Korea; jeoung66@kaya.ac.kr; 3 Department of Global Health and Disease Control, Yonsei University, Seoul 03722, Republic of Korea; ddchapagain@gmail.com; 4 Department of Public Health, Ministry of Health, Hetauda 44107, Bagmati Province, Nepal; 5 Department of Biohealth Industry, Graduate School of Transdisciplinary Health Science, Yonsei University, Seoul 03722, Republic of Korea; thkim@yuhs.ac; 6 Department of Preventive Medicine, College of Medicine, Yonsei University, Seoul 03722, Republic of Korea; leevan@yuhs.ac

**Keywords:** low-carbohydrate diet, metabolic syndrome, macronutrient intake, cardiometabolic risk, Korean adults, dietary patterns, obesity, clinical nutrition

## Abstract

**Aims:** Low-carbohydrate diets (LCDs) are associated with metabolic benefits, but their long-term effects remain uncertain, particularly in Asian populations with traditionally high carbohydrate intake. This study examined LCD patterns and their association with metabolic syndrome (MetS) in Korean adults using nationally representative data from the 2022–2023 Korea National Health and Nutrition Examination Survey (KNHANES). **Methods:** Among 9617 adults aged ≥20 years with complete dietary and health data, LCD score was calculated from the percentage of energy derived from carbohydrates (reverse-scored), fats, and proteins, and participants were categorized into high-carbohydrate, moderate-carbohydrate, and low-carbohydrate groups. MetS was defined using an Adult Treatment Panel III and Korean criteria. Survey-weighted logistic regression was applied to assess associations between LCD score and MetS across sequentially adjusted models. **Results:** MetS prevalence differed significantly across LCD decile groups (LCD1: 9.6%, LCD2: 5.8%, LCD3: 9.7%; *p* < 0.001). In a minimally adjusted model, LCD decile 3 was associated with higher odds of MetS (OR, 1.14; 95% CI: 1.02–1.27). However, this association was attenuated and became non-significant after further adjustment for key metabolic risk factors. Obesity, blood pressure, fasting glucose, triglycerides, and high-density lipoprotein cholesterol were all strongly associated with MetS (all *p* < 0.001), and income-related disparities were evident, with lower-income groups showing higher carbohydrate and lower fat and protein intakes. **Conclusions:** These findings suggest that LCD patterns are not independently associated with MetS once underlying metabolic factors are considered. Public health strategies in Korea would be emphasized by improving nutrient quality, promoting balanced macronutrient intake, and reducing socioeconomic inequalities in diet to mitigate metabolic risk among adults.

## 1. Introduction

Metabolic syndrome (MetS) has emerged as one of the most pressing public health challenges worldwide, characterized by abdominal obesity, dyslipidemia, impaired glucose regulation, and elevated blood pressure [1]. These metabolic abnormalities significantly increase the risk of type 2 diabetes, cardiovascular disease, and all-cause mortality [2]. Although MetS is a global health challenge, the dietary and lifestyle factors driving its development differ markedly across populations [3]. Evidence from North America and Europe suggested that diets lower in carbohydrates and higher in unsaturated fats improve lipid profiles, reduce hepatic fat accumulation, and enhance glycemic control [4,5]. However, global findings are heterogeneous, and results from Western countries may not directly translate to Asian populations, where carbohydrate consumption levels, meal patterns, and metabolic phenotypes differ substantially.

Low-carbohydrate diets (LCDs) have gained international attention due to their favorable effects on weight loss, insulin sensitivity, and cardiometabolic risk biomarkers [6,7]. In comparison to low-fat diets, large, randomized trials have shown that LCDs can produce similar or better metabolic improvements in a variety of populations [8,9,10]. However, Asian populations, particularly East Asians, exhibit a distinct cardiometabolic phenotype marked by a greater proclivity for visceral fat storage, insulin resistance, and metabolic abnormalities, even at lower BMI levels [3,11]. This distinct phenotype raises the question of whether LCDs exert similar or stronger benefits in Asian contexts relative to Western populations.

According to comparative findings from Japan, China, and Singapore, Asians build visceral adipose tissue faster than Caucasians at the same BMI, resulting in higher triglycerides and worse glucose control [12,13,14,15,16]. Therefore, it is still unclear if LCDs provide comparable or perhaps higher metabolic benefits in Asian populations when compared to their Western populations [17,18]. Supporting this hypothesis, studies in Chinese and Japanese adults suggest that reducing carbohydrate intake even modestly can improve lipid ratios, insulin resistance indices, and hepatic fat content more substantially than in Western cohorts [11,14]. In South Korea, carbohydrates have traditionally contributed more than 60–70% of total energy intake, one of the highest proportions among OECD countries, primarily due to the predominance of white rice in the diet [19]. Recent Korean epidemiologic studies suggest that high-carbohydrate diets are associated with increased risks of hypertriglyceridemia, reduced HDL cholesterol, and central obesity, even after adjustment for lifestyle and socioeconomic confounders [20,21,22]. Korea’s shift toward refined grains and processed foods has gone hand in hand with an increase in metabolic health problems. At the same time, dietary gaps between socioeconomic groups have widened, with lower-income households more likely to depend on cheap, carbohydrate-heavy foods and to have less diverse, lower-quality diets, creating complex patterns of diet quality across income levels.

Despite this broader context, research on low-carbohydrate diets in Korea is still relatively scarce and often relies on older KNHANES data, narrow macronutrient-focused analyses, or diet measures that do not use internationally recognized quality scoring methods. Few studies have looked at how sticking to a low-carbohydrate diet overlaps with socioeconomic differences or clusters with metabolic markers like triglycerides, HDL cholesterol, visceral fat, and glucose control.

This study examines how a low-carbohydrate diet score relates to metabolic syndrome risk in Korean adults using the latest nationally representative 2022–2023 KNHANES data, incorporating survey weights and comparisons across income groups. The results can help refine Korea’s dietary guidelines, support more targeted prevention strategies, and add to global knowledge on how macronutrient balance shapes metabolic risk in specific populations.

## 2. Materials and Methods

### 2.1. Study Design and Data Source

This cross-sectional study used data from the 2022–2023 Korea National Health and Nutrition Examination Survey (KNHANES), a nationally representative survey conducted annually by the Korea Disease Control and Prevention Agency (KDCA). KNHANES employs a rolling sampling design with multistage, stratified, cluster probability sampling based on geographic area, sex, and age distribution to ensure representativeness of the civilian Korean population. All participants provided written informed consent, and the survey received ethical approval from the KDCA institutional review board. The present analysis included adults aged ≥20 years who completed the health interview, health examination, and 24-h dietary recall components and had no missing data for primary exposure, outcome, or covariates.

### 2.2. Variables

#### 2.2.1. Assessment of Dietary Intake and Low-Carbohydrate Diet Score

Dietary intake was assessed using a standardized 24-h recall interview conducted by trained KNHANES dietitians. Nutrient intake and energy contribution from carbohydrates, proteins, and fats were calculated using the Korean Food Composition Table. A LCD score was constructed following Halton et al.’s method [23], using sex-specific deciles of percentage energy from carbohydrates (reverse-scored), fats, and proteins (positively scored). The three components were summed to produce a score ranging from 3 to 30, with higher values indicating greater adherence to a low-carbohydrate diet. For analysis, the LCD score was categorized into three groups: high-carbohydrate (Decile 1), moderate (Decile 2), and low-carbohydrate intake (Decile 1 to 3). This categorization reflects increasing adherence to an LCD pattern across groups LCD1 = highest carbohydrate intake, LCD2 = moderate carbohydrate intake, and LCD3 = lowest carbohydrate intake.

#### 2.2.2. Assessment of Metabolic Syndrome

MetS was defined using the NCEP–ATP III criteria modified for Asian populations. Participants were classified as having MetS if they met three or more of the following components: (1) abdominal obesity: waist circumference ≥90 cm for men or ≥80 cm for women; (2) elevated blood pressure: systolic ≥130 mmHg, diastolic ≥85 mmHg, or antihypertensive medication use; (3) elevated triglycerides: ≥150 mg/dL; (4) low HDL-cholesterol: <40 mg/dL in men or <50 mg/dL in women; and (5) elevated fasting glucose: ≥100 mg/dL or use of diabetes medication. All measurements were obtained during the KNHANES health examination by trained personnel using standardized protocols.

#### 2.2.3. Covariates

Sociodemographic variables included age group (20–39, 40–49, 50–64, and ≥65 years), sex (male, female), education level (Elementary, Middle, High and college and over), and residential area (urban vs. rural). Monthly household income was determined by summing up the earnings of all family members, including any external financial assistance. Smoking behavior was categorized as either smokers (current or occasional) or non-smokers (former or never). Alcohol intake was classified into drinkers (current) and non-drinkers (past or never). Physical activity was assessed in metabolic equivalent hours per week (MET-h/week) and divided into two categories: Yes (regular) and No (Irregular) according to tertiles of the distribution. Clinical related metabolic indicators variables included body mass index (BMI), systolic and diastolic blood pressure, fasting glucose, triglycerides, HDL-cholesterol, and LDL-cholesterol, all measured during the health examination. These variables were modeled as continuous covariates in the fully adjusted model to account for underlying cardiometabolic status and to minimize residual confounding when estimating the independent association of low-carbohydrate dietary patterns with MetS risk.

### 2.3. Statistical Analysis

All analyses incorporated the complex survey design of KNHANES using sampling weights, stratification, and clustering to ensure national representativeness. Descriptive statistics were presented as weighted means ± standard errors for continuous variables and weighted frequency and percentages for categorical variables, stratified by LCD categories. Group differences in dietary intake and health indicators across LCD categories and income quartiles were evaluated using survey-weighted ANOVA for continuous variables and Rao–Scott χ^2^ tests for categorical variables. Survey-weighted logistic regression models were used to assess the association between LCDs and metabolic syndrome. Three hierarchical models were constructed: Model 1 (Unadjusted), Model 2 (adjusted for sociodemographic factors, lifestyle behaviors, and clinical indicators including BMI). Adjusted odds ratios (ORs) with 95% confidence intervals were reported. Effect modification by sex was evaluated using interaction terms and sex-stratified models. Model fit was evaluated using AIC, and discrimination was assessed using the C-statistic (AUC). Statistical significance was defined as *p* < 0.05, and analyses were performed using SAS 9.4 (SAS Institute, Cary, NC, USA).

## 3. Results

### 3.1. Low-Carbohydrate-Diet Score and Percentage of Total Daily Energy

**Figure 1:**
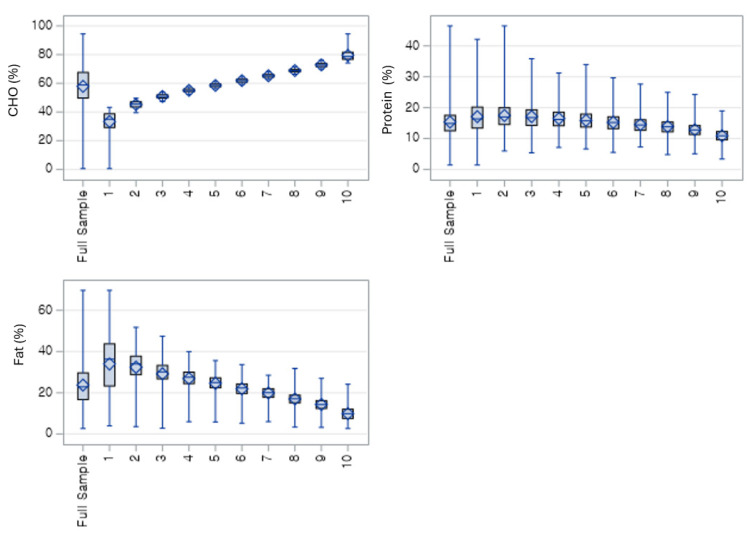
Weighted distribution of macronutrient energy contribution (%) across carbohydrate intake deciles. Carbohydrate percentage increases steadily from decile 1 to 10, while fat percentage decreases correspondingly; protein percentage remains relatively stable with a slight downward trend. CHO = % of total energy from carbohydrates; Protein = % of total energy from protein; FAT = % of total energy from fat.

Figure 1 shows the expected macronutrient trends across the 10 deciles of the low-carbohydrate diet (LCD) score. Carbohydrate intake (%) declines steadily from Decile 1 to Decile 10, indicating progressively lower carbohydrate consumption among individuals with higher LCD scores. In contrast, fat intake (%) increases consistently across declines, reflecting the compensatory rise in dietary fat as carbohydrate intake decreases. Protein intake shows a more modest downward trend, with greater variability across deciles compared with carbohydrates and fats. The category of the LCD 2 and 3 with individuals tend to have younger age, slightly lower systolic and diastolic blood pressure, and lower fasting glucose, compared with the full sample and the high-carbohydrate group (Supplementary Figure S1).

### 3.2. Comparison of the Mets Category and LCD Types with Covariates

Table 1 presents the weighted baseline characteristics of Korean adults across low-carbohydrate diet categories. Among the 9617 participants, metabolic syndrome prevalence was highest in the LCD decile 3 group (9.7%) than other deciles. Younger adults aged 20–39 years were much more common in the low-carbohydrate group (15.1%) than other decile groups. Educational attainment of elementary education decreasing from the high-carbohydrate group (9.9%) to the low-carbohydrate group (3.7%), and the distribution of lowest-income individuals showing a similar trend (8.8% vs. 10.2%). Normal BMI showed a high prevalence of LCD3 (16.1%) than other groups. Metabolic indicators of waist circumference were greatest in the decile1 group (84.87 cm); fasting glucose was highest in the high-carbohydrate group (102.77 mg/dL) and lowest in the low-carbohydrate group (98.62 mg/dL); triglyceride levels followed the same pattern (134.02 mg/dL vs. 125.26 mg/dL). HDL-cholesterol increased progressively from the high-carbohydrate group (54.70 mg/dL) to the low-carbohydrate group (58.45 mg/dL).

### 3.3. Multivariate Logistic Regression Models for Association of LCD and Covariates

Table 2 presents the logistic regression models to find the association between LCD categories and MetS among Korean adults. In Model 1, individuals in the lowest carbohydrate intake group showed a modest but statistically significant higher odds of metabolic syndrome compared with the highest intake group (OR = 1.14, 95% CI: 1.01–1.26, *p* = 0.02), while the moderate group did not differ significantly (OR = 0.98, *p* = 0.50). This association persisted after multivariable adjustment in Model 2 (aOR = 1.15, 95% CI: 1.05–1.40, *p* = 0.02). Increasing age remained consistently associated with higher odds of metabolic syndrome in both models (*p* < 0.001). In the adjusted model, females had significantly lower odds (aOR = 0.32, 95% CI: 0.27–0.38, *p* < 0.001) compared with males. Metabolic risk components were strongly associated with MetS in the expected directions, including higher BMI, SBP, DBP, glucose, and triglycerides (all *p* < 0.001), while higher HDL cholesterol was protective (aOR = 0.91, *p* < 0.001); LDL cholesterol was not significantly associated (*p* = 0.65).

### 3.4. Association Between the MetS with Covariates and Metabolic Predictors

**Figure 2:**
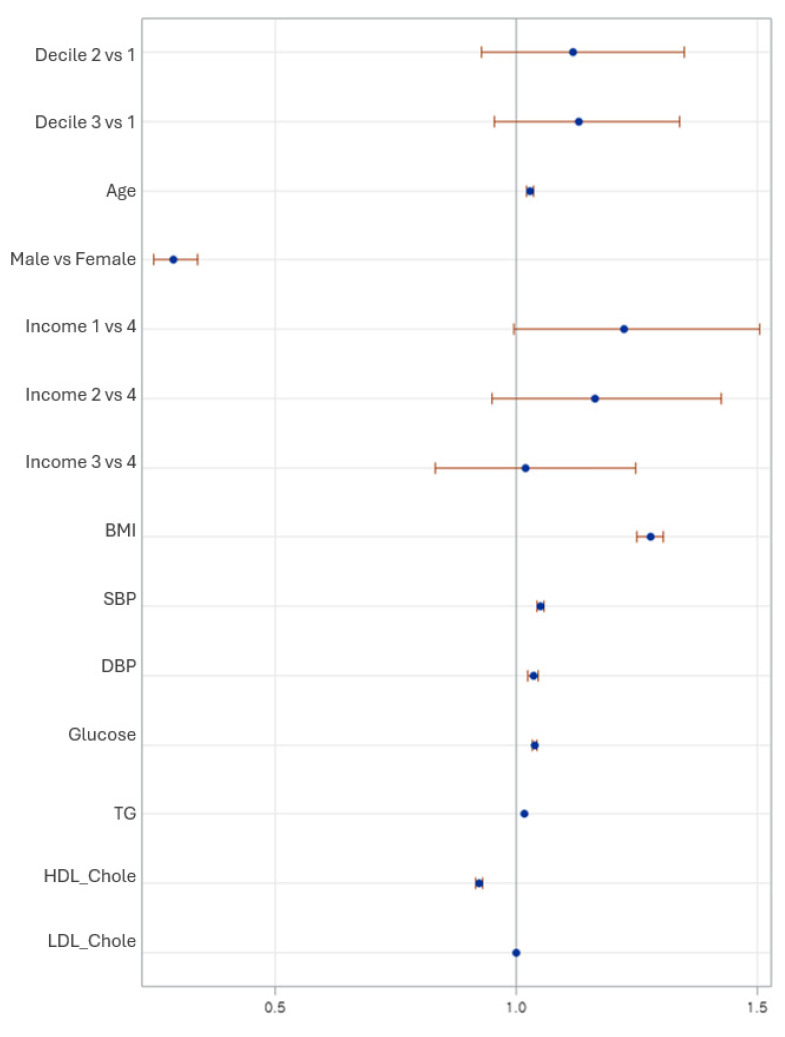
Forest plot showing adjusted odds ratios (OR) and 95% confidence intervals for metabolic syndrome based on low-carbohydrate diet deciles and covariates. Estimates are derived from the fully adjusted survey-weighted logistic regression model (Model 2), accounting for age, sex, household income, smoking, alcohol intake, and physical activity, as well as BMI and metabolic indicators.

Figure 2 shows the adjusted odds ratios for metabolic syndrome across key predictors. the LCD3 had significantly higher odds of MetS (OR = 1.15, 95% CI: 1.05–1.40), whereas no significant association found in LCD2 (OR = 1.15, 95% CI: 0.92–1.44) compared than LCD1. The older age, high BMI, systolic and diastolic blood pressure, fasting glucose, and triglycerides were all strongly associated with increased MetS risk, whereas HDL-cholesterol was protective (OR = 0.92). Lower-income adults showed higher odds of MetS, and women had significantly lower risk than men.

#### Sex-Stratified Association Between MetS and LCDs Group with Covariates

Table 3 shows the sex-stratified multivariable associations between low-carbohydrate diet categories and metabolic syndrome. Among men, neither moderate (AOR = 0.98; 95% CI: 0.59–1.61; *p* = 0.92) nor low carbohydrate intake (AOR = 1.02; 95% CI: 0.68–1.53; *p* = 0.93) was significantly associated with metabolic syndrome; however, age (AOR = 1.01; 95% CI: 1.00–1.03; *p* = 0.03) and smoking (AOR = 1.42; 95% CI: 1.05–1.92; *p* = 0.02) significantly increased risk. Among women, carbohydrate intake similarly showed no significant associations (LCD2 AOR = 1.41; 95% CI: 0.44–4.51; *p* = 0.57; LCD3 AOR = 1.71; 95% CI: 0.61–4.84; *p* = 0.31); however, age showed a strong positive association (AOR = 1.08; 95% CI: 1.05–1.12; *p* < 0.001), and women in the lowest income group had a markedly increased risk (AOR = 4.36; 95% CI: 1.33–14.36; *p* = 0.02). Other behavioral factors, including alcohol and physical activity, were not significant.

### 3.5. Subgroup Analysis

#### 3.5.1. Macronutrient Intake by Income Level

Table 4 exhibits the socioeconomic differences in macronutrient intake across the four income groups, Participants in the lowest-income had the highest carbohydrate intake (60.60 ± 13.50% of energy), which steadily declined across higher-income groups, reaching 57.85 ± 12.98% in the highest-income category (*p* < 0.0001). In contrast, fat intake showed the opposite pattern, increasing progressively from 21.69 ± 9.60% in the lowest-income group to 24.13 ± 9.63% in the highest-income group (*p* < 0.0001). Protein intake also increased slightly with higher income, rising from 14.87 ± 4.18% to 15.50 ± 4.04% across the income gradient (*p* < 0.0001).

Adults in the lowest-income group had a 36% likely to increase in MetS compared with the highest-income group. Mid-income groups showed no significant difference after full adjustment. Meanwhile, nutrient variables (CHO, FAT, PROT %) and metabolic factors explain much of the socioeconomic disparity (Table 5).

#### 3.5.2. Prediction of Metabolic Syndrome Among Adults in the LCD Group

**Figure 3:**
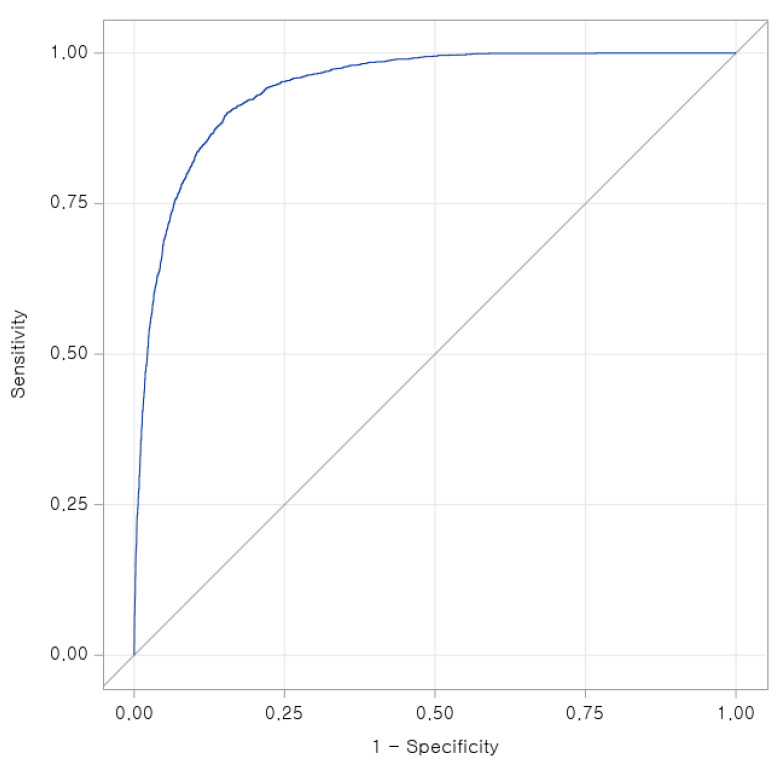
Receiver operating characteristic (ROC) curve for predicting metabolic syndrome among adults in the lowest-carbohydrate intake group (LCD Decile 3).

The ROC curve shows excellent model discrimination, with a steep rise toward the upper-left corner indicating high sensitivity at low false-positive rates. The area under the curve (AUC = 0.94) demonstrates strong predictive accuracy, confirming that the combined model including LCD category, demographic factors, and metabolic indicators effectively differentiates individuals with and without metabolic syndrome (Figure 3).

## 4. Discussion

This nationally representative analysis of Korean adults from KNHANES 2022–2023 demonstrates that LCD patterns are closely linked to MetS risk, dietary macronutrient distributions, and socioeconomic gradients. As Korea undergoes a rapid nutritional transition from a rice-centered, high-carbohydrate pattern toward more Westernized eating behaviors, understanding how macronutrient composition affects metabolic health is increasingly important. Our findings indicate that higher adherence to LCDs, characterized by greater carbohydrate restriction, is associated with a higher prevalence of MetS. This contrasts with conclusions derived from numerous clinical trials but is consistent with emerging evidence from Asian cohort studies indicating that carbohydrate quality, fat type, and protein source significantly influence the metabolic outcomes of LCD deciles [21,24]. Korea continues to have one of the highest carbohydrate intakes globally, particularly from refined grains such as white rice [25], which may explain why metabolic responses to carbohydrate reduction differ from Western populations consuming higher-fat baselines.

In adjusted logistic models, adults in the LCD 3 had 15–20% higher odds of MetS, even after controlling for age, sex, income, obesity, lipids, and blood pressure. This finding is consistent with multi-country evidence from the PURE study, which reported that diets very low in carbohydrates particularly when replaced by animal fats and proteins were associated with higher total mortality and cardiometabolic risk [26]. Similarly, the Atherosclerosis Risk in Communities (ARIC) study found U-shaped associations, where both very low and very high carbohydrate intake increased mortality risk due to differential substitutions [27,28]. In the Korean context, low-carbohydrate diets commonly substitute rice with processed meats, barbeque-style dishes, or animal-fat–rich meals, unlike Mediterranean or Japanese LCDs that emphasize fish, vegetables, and unsaturated fats [29,30]. This quality gap likely contributes to the adverse associations observed.

Socioeconomic differences in macronutrient intake stood out clearly. Low-income adults ate significantly more carbohydrates and less fat and protein, driven by economic barriers that limit food access and choices. Similar patterns appear in U.S. NHANES data and European studies [18,31]. In Korea, cheaper, energy-dense grain staples dominate low-income diets, while higher-income households enjoy more varied options like dairy, meat, and nuts [32,33]. These gaps help explain why low-income groups face higher odds of metabolic syndrome and poor metabolic markers. The results echo Korean research calling for targeted nutrition programs for vulnerable populations [34]. Thus, macronutrient proportion alone may not adequately reflect metabolic risk; rather, diet quality, food diversity, and nutrient balance play a more decisive role. Low-income groups tend to experience poorer overall diet quality, limited access to high-quality protein and healthy unsaturated fats, and greater reliance on inexpensive, nutrient-poor foods, which cumulatively increase cardiometabolic vulnerability.

Our findings reveal meaningful rural–urban differences in dietary patterns and metabolic risk in Korea. Urban adults were more likely to follow lower-carbohydrate dietary patterns, reflecting greater food diversity and shifting lifestyle behaviors, whereas rural adults continued to rely more heavily on carbohydrate-dominant diets [10] such as refined rice. Similar trends have been reported previously, showing that urbanization in Korea and other Asian populations is linked to greater dietary variety and higher intake of protein and fat, while rural areas often face limited access to nutrient-dense foods and remain at higher risk of metabolic abnormalities [19,25]. Studies have also shown socioeconomic and geographic dietary inequalities contributing to central obesity, dyslipidemia, and glucose abnormalities in rural residents despite comparable or lower BMI [21,28]. These updated results reinforce that dietary transitions in Korea are not uniform and highlight the need for region-specific nutrition strategies that ensure healthier food environments and equitable access to quality foods across both rural and urban communities.

The predictive discrimination for AUC suggests, MetS when combining dietary, demographic, and metabolic indicators. The strong contributions of obesity, triglycerides, fasting glucose, and HDL-cholesterol mirror evidence from Asian metabolic risk algorithms, which highlight the heightened cardiometabolic sensitivity to visceral adiposity and insulin resistance among East Asian populations [12,35,36]. Furthermore, our domain analysis confirmed that as LCD score increased, carbohydrate intake steadily declined while fat intake rose, reflecting consistent internal validity of the LCD score. Given that Koreans may be particularly insulin-sensitive and predisposed to dyslipidemia at lower BMI thresholds [37], even small dietary shifts toward high-fat, low-carbohydrate patterns may disproportionately elevate MetS risk.

The findings of this study are particularly relevant to the Korean context, where dietary intake remains predominantly carbohydrate-based, largely driven by refined rice consumption, which differs from many Western dietary environments. Moreover, Koreans and other East Asian populations exhibit a distinct metabolic phenotype characterized by higher visceral adiposity, insulin resistance, and cardiometabolic risk even at lower BMI levels, potentially reflecting both genetic susceptibility and diet–gene interactions. These population-specific dietary and biological characteristics suggest that the observed associations may be especially meaningful for Koreans and other East Asian groups, although further cross-ethnic studies are needed to confirm generalizability. In addition, it also aligns with the growing body of evidence cautioning against unregulated, Western-style ketogenic or extreme low-carb diets in East Asian populations. Several recent studies in Japan, Taiwan, and China have reported that high animal-fat LCDs exacerbate LDL cholesterol, hepatic fat accumulation, and systemic inflammation [11,14]. Conversely, moderate LCDs emphasizing plant-based fats, fish, legumes, and low-glycemic carbohydrates tend to improve metabolic profiles [38]. Thus, the composition not simply the proportion of macronutrients remains a key determinant of metabolic outcomes.

Overall, our findings highlight a critical public health message: not all LCDs are metabolically beneficial, and culturally inappropriate versions may worsen MetS in Korean adults. Given the rapid dietary Westernization and persistent socioeconomic inequalities, national dietary guidelines should emphasize balanced macronutrient patterns, replacing refined carbohydrates with whole grains, fruits, nuts, fish, and unsaturated fats, rather than indiscriminately reducing carbohydrates. Previous long-term trials of LCDs have generally evaluated and libitum intake without fixed caloric restriction [39,40]. While this approach reflects real-world self-selected dietary behavior, it makes it difficult to isolate the independent effects of LCDs on cardiometabolic outcomes due to confounding from variable energy intake [41]. Moreover, many of these studies report high dropout rates, poor long-term adherence, and only modest sustained weight loss [42]. Consequently, although LC diets have gained widespread public interest, uncertainty remains regarding their long-term efficacy and specific cardiometabolic effects independent of energy intake and weight change [43]. Longitudinal cohort studies are needed to determine the long-term metabolic effects of culturally adapted low-carbohydrate dietary patterns and to assess dietary quality as a moderating factor. Ultimately, precision nutrition strategies tailored to Korean dietary habits and metabolic phenotypes may offer the most effective approach for preventing metabolic syndrome.

The findings highlight the need for tailored nutritional guidelines in Korea that go beyond macronutrient proportions and emphasize dietary quality, including whole grains, legumes, vegetables, and unsaturated fat sources instead of simply reducing carbohydrate intake. Public health strategies should incorporate socioeconomic disparities, as lower-income groups displayed more carbohydrate-dominant diets and higher metabolic risk. Policies promoting food affordability, access to healthier ingredients, school- and workplace-based dietary education, and subsidies for nutrient-dense foods may help reduce equity gaps. Moreover, integrating personalized nutrition counseling into national health screenings could support individuals at high metabolic risk. Collectively, these measures can strengthen Korea’s chronic disease prevention frameworks amid rapid dietary transitions.

### 4.1. Limitations

This study has several limitations that should be acknowledged. First, its cross-sectional design prevents the establishment of causal relationships between low-carbohydrate diet patterns and metabolic syndrome, and diet–disease associations may be influenced by reverse causality. Second, dietary intake was assessed via a single 24-h recall, which may not fully capture habitual intake or long-term dietary patterns and is subject to recall bias. Although survey weighting improves representativeness, unmeasured confounding such as dietary quality, physical activity details, genetic predisposition, or cooking methods may still influence the observed associations. Additionally, the LCD score used here does not distinguish between plant-based versus animal-based fat and protein sources, which may have differential metabolic impacts. Finally, findings are specific to Korean adults and may not generalize to other populations with different dietary cultures or metabolic phenotypes.

### 4.2. Policy Implications

These findings suggest that Korean dietary policy should move beyond focusing on macronutrient ratios alone and place greater emphasis on overall diet quality. Rather than simply encouraging people to “eat fewer carbohydrates”, policies should promote healthier carbohydrate and fat choices, such as whole grains, legumes, vegetables, fruits, and unsaturated fats. Public health efforts must also take socioeconomic differences into account, as lower-income groups tended to rely more heavily on carbohydrate-based diets and showed greater metabolic risk. National strategies could therefore include subsidies or price-support programs for nutrient-dense staple foods, improved access to healthier options in traditional markets and convenience stores, expanded nutrition programs in schools and workplaces, and culturally sensitive nutrition education that reflects typical Korean meal patterns. In addition, incorporating personalized nutrition counseling into national health screenings and primary care, especially for individuals with early signs of metabolic problems, could help support long-term dietary changes. Strengthening these measures within Korea’s existing chronic disease prevention framework may help reduce metabolic risk in the context of rapid dietary change and an evolving food environment.

## 5. Conclusions

This nationally representative study of Korean adults demonstrates that lower-carbohydrate dietary patterns were associated with metabolic syndrome risk, but these relationships were strongly modified by age, socioeconomic status, and baseline metabolic health. Individuals with higher carbohydrate intake tended to be older and to exhibit less favorable metabolic profiles; however, the initially elevated MetS risk observed among low-carbohydrate consumers was attenuated after adjustment for key metabolic indicators, highlighting the complexity of diet–metabolic interactions and the potential for overadjustment. These findings suggest that carbohydrate quantity alone may be less important than overall diet quality, nutrient balance, and population-specific metabolic characteristics. Moreover, socioeconomic disparities markedly shaped dietary patterns, underscoring the importance of equitable access to healthier food environments. Collectively, the results support the need for Korean dietary guidance that integrates macronutrient composition with cultural dietary norms, metabolic vulnerability, and social determinants of nutrition. Further longitudinal and intervention studies are warranted to clarify causality, define optimal carbohydrate thresholds for Asian populations, and inform precision nutrition strategies for MetS. Moreover, income-related disparities in nutrient intake highlight the urgent need for affordability and accessibility policies such as healthier food subsidies, taxation of ultra-processed foods, and targeted nutrition support programs for lower-income groups. Together, these nutrient-focused and equity-oriented policies may help Korea more effectively mitigate the rising burden of metabolic syndrome and cardiometabolic diseases.

## Figures and Tables

**Table 1:** General characteristics of the participants with Low carbohydrate diet intake categories by decile.

Variables	Carbohydrate Diet Intake						*p*
	Decile 1		Decile 2		Decile 3		
	(High)		(Moderate)		(Low)		
	N	%	N	%	N	%	
Total	3000	31.2	2110	21.9	4507	46.9	
MetS							<0.001
No	2080	21.6	1557	16.2	3572	37.1	
Yes	920	9.6	553	5.8	935	9.7	
Age (Years)	56.35 ± 0.46		49.45 ± 0.48		44.73 ± 0.34		<0.001
Age group (Years)							0.048
20–39	322	3.3	437	4.5	1456	15.1	
40–49	328	3.4	350	3.6	942	9.8	
50–64	917	9.5	707	7.4	1290	13.4	
≥65	1433	14.9	616	6.4	819	8.5	
Sex							<0.001
Male	1282	13.3	960	10	1964	20.4	
Female	1718	17.9	1150	12	2543	26.4	
Education							<0.001
Elementary or less	950	9.9	306	3.2	354	3.7	
Middle school	403	4.2	217	2.3	291	3	
High school	909	9.5	746	7.8	1523	15.8	
College or higher	738	7.7	841	8.7	2339	24.3	
Income							<0.001
Lowest	851	8.8	534	5.6	979	10.2	
Low-middle	803	8.3	527	5.5	1096	11.4	
High-middle	729	7.6	523	5.4	1196	12.4	
Highest	617	6.4	526	5.5	1236	12.9	
Residence							
Urban	2134	22.2	1664	17.3	3775	39.3	<0.001
Rural	866	9	446	4.6	732	7.6	
Smoking							<0.001
Yes	1658	17.2	1060	11.0	1769	18.4	
No	1342	14.0	1050	10.9	1638	17.0	
Alcohol consumption							0.001
Yes	2064	21.5	1400	14.6	2546	26.5	
No	936	9.7	710	7.4	1961	20.4	
Physical activity							0.002
Yes	2506		1765	18.4	3725	38.7	
No	494		345	3.6	782	8.1	
BMI Categories							0.046
Underweight	106	1.1	110	1.1	398	4.1	
Normal	1041	10.8	727	7.6	1628	16.9	
Overweight	678	7.1	495	5.1	925	9.6	
Obese	983	10.2	640	6.7	1258	13.1	
Very obese	192	2	138	1.4	298	3.1	
Obesity and Metabolic variables (M ± SD)							
Waist circumference (cm)	84.87 ± 0.24		84.51 ± 0.30		83.39 ± 0.21		<0.001
SBP (mmHg)	120.93 ± 0.40		118.92 ± 0.41		116.27 ± 0.29		<0.001
DBP (mmHg)	74.44 ± 0.24		74.67 ± 0.26		73.51 ± 0.19		<0.001
BMI (kg/m^2^)	24.21 ± 0.08		24.29 ± 0.11		24.18 ± 0.07		0.004
Fasting glucose (mg/dL)	102.77 ± 0.58		99.99 ± 0.50		98.62 ± 0.37		0.021
Triglycerides (mg/dL)	134.02 ± 2.17		133.15 ± 2.69		125.26 ± 1.70		0.040
HDL-cholesterol (mg/dL)	54.70 ± 0.33		56.57 ± 0.35		58.45 ± 0.28		<0.001
LDL-cholesterol (mg/dL)	112.24 ± 0.90		115.51 ± 1.06		117.35 ± 0.60		0.166

MetS, Metabolic syndrome; N, number; %, Survey-weighted percentage; M ± SD, Mean and standard deviation; BMI, Body mass index; SBP, Systolic blood pressure; DBP, Diastolic blood pressure; HDL, High-density lipoprotein; LDL, Low-density lipoprotein.

**Table 2:** Association models between Outcomes variables and Metabolic Syndrome.

Variables	Metabolic Syndrome (Yes)					
	Model 1 ^a^			Model 2 ^b^		
	OR	95% CI	*p*	aOR	95% CI	*p*
Low carbohydrate diet intake						
Decile 1 (High)	1.00			1.00		
Decile 2 (Medium)	0.98	0.84–1.16	0.50	1.15	0.92–1.44	0.49
Decile 3 (Low)	1.14	1.01–1.26	0.02	1.15	1.045–1.40	0.02
Age (Years)	1.03	1.02–1.06	<0.001	1.03	1.022–1.04	<0.001
Sex						
Male				1.00		
Female				0.32	0.266–0.381	<0.001
BMI & Metabolic Indicators						
BMI				1.25	1.22–1.29	<0.001
SBP				1.05	1.04–1.05	<0.001
DBP				1.04	1.02–1.05	<0.001
Glucose				1.04	1.028–1.04	<0.001
Triglycerides				1.01	1.012–1.02	<0.001
HDL-cholesterol				0.91	0.910–0.92	<0.001
LDL-cholesterol				0.99	0.99–1.02	0.65

^a^ Model 1, Unadjusted OR for Association between the Low carbohydrate diet and metabolic syndrome risk without adjusting covariates and ^b^ Model 2, Adjusted with covariates and clinical indicators; BMI—Body mass index; SBP—Systolic blood pressure; DBP—Diastolic blood pressure; HDL—High-density lipoprotein; LDL—Low-density lipoprotein; CI, confidence interval.

**Table 3:** Sex-stratified association between MetS and LCD decile with socioeconomic covariates.

	Metabolic Syndrome (Yes)					
Variable	Male			Female		
	AOR	95% CI	*p*	AOR	95% CI	*p*
Carbohydrate diet intake						
Decile 1 (High)	1.00					
Decile 2 (Medium)	0.98	0.59–1.61	0.92	1.41	0.44–4.51	0.57
Decile 3 (Low)	1.02	0.68–1.53	0.93	1.71	0.61–4.84	0.31
Age (Years)	1.01	1.00–1.03	0.03	1.08	1.05–1.12	<0.001
Income						
Lowest	1.47	0.88–2.46	0.14	4.36	1.33–14.36	0.02
Low-middle	1.31	0.79–2.17	0.30	0.66	0.14–3.01	0.59
High-middle	1.02	0.59–1.75	0.94	0.76	0.17–3.50	0.73
Highest	1.00					
Smoking						
Yes	1.42	1.05–1.92	0.02	0.59	0.26–1.34	0.20
No	1.00					
Alcohol use						
Yes	0.73	0.51–1.07	0.10	1.51	0.60–3.82	0.38
No	1.00					
Physical activity						
Yes	0.94	0.58–1.52	0.80	0.60	0.17–2.13	0.43
No	1.00					
Clinical Metabolic indicators						
Waist circumference	0.77	0.76–0.78	<0.001	1.31	1.29–1.32	<0.001
SBP	1.01	1.01–1.02	<0.001	0.99	0.99–0.99	<0.001
DBP	0.95	0.94–0.95	<0.001	1.06	1.05–1.07	<0.001
Glucose	1.00	1.00–1.00	0.07	1.00	1.00–1.01	0.073
Triglycerides	1.00	0.99–1.00	0.23	1.00	1.00–1.01	0.231
HDL-cholesterol	1.05	1.05–1.06	<0.001	0.95	0.95–0.96	<0.001
LDL-cholesterol	1.00	1.00–1.01	0.14	1.00	0.99–1.00	0.138

MetS, Metabolic syndrome; LCD, Low-carbohydrate diet; AOR, Adjusted with education, insurance, residence and body mass index; CI, confidence interval; SBP—Systolic blood pressure; DBP—Diastolic blood pressure; HDL—High-density lipoprotein; LDL—Low-density lipoprotein.

**Table 4:** Association between the income level and macronutrient intake.

Variable	Lowest	Low-Middle	High-Middle	Highest	*p*
CHO intake	60.60 ± 13.50	59.49 ± 13.58	59.13 ± 12.83	57.85 ± 12.98	<0.001
Fat intake	21.69 ± 9.60	22.56 ± 9.86	22.88 ± 9.38	24.13 ± 9.63	<0.001
Protein intake	14.87 ± 4.18	15.06 ± 4.09	15.39 ± 4.10	15.50 ± 4.04	<0.001

CHO, carbohydrate.

**Table 5:** Macronutrient intake by income level adjusted by covariates.

Predictor	Adjusted OR	95% CI	*p*
Income 1 (Lowest)	1.366	1.132–1.649	<0.001
Income 2 (Low-middle)	1.138	0.939–1.381	0.189
Income 3 (High-middle)	1.011	0.836–1.223	0.908
Income 4 (Highest)	1.000		

## Data Availability

The datasets used in this study are publicly available in Korea National Health and Nutritional Examination Survey (KNHANES) data archives: https://knhanes.kdca.go.kr/knhanes/main.do# (accessed on 15 October 2025).

## Supplementary Materials

The following supporting information can be downloaded at: https://www.mdpi.com/article/10.3390/nu18010178/s1. Figure S1: Distribution of demographic and metabolic characteristics across full sample of low-carbohydrate diet (LCD) groups.

## Abbreviations

The following abbreviations are used in this manuscript:

BMI	Body Mass Index
BP	Blood Pressure
DBP	Diastolic Blood Pressure
FPG	Fasting Plasma Glucose
HDL	High-Density Lipoprotein Cholesterol
KNHANES	Korea National Health and Nutrition Examination Survey
LCD	Low-Carbohydrate Diet
LCD_dec	Low-Carbohydrate Diet Score Deciles
LDL	Low-Density Lipoprotein Cholesterol
MetS	Metabolic Syndrome
SBP	Systolic Blood Pressure
TG	Triglycerides
