# Supplementary figures and images for: Low-Carbohydrate Diet and Metabolic Syndrome Risk in Korean Adults: A Korea National Health and Nutrition Examination Survey Study
Source: Nutrients. 2026 Jan 5;18(1):178. doi: 10.3390/nu18010178 (PMC12787864; doi:10.3390/nu18010178)

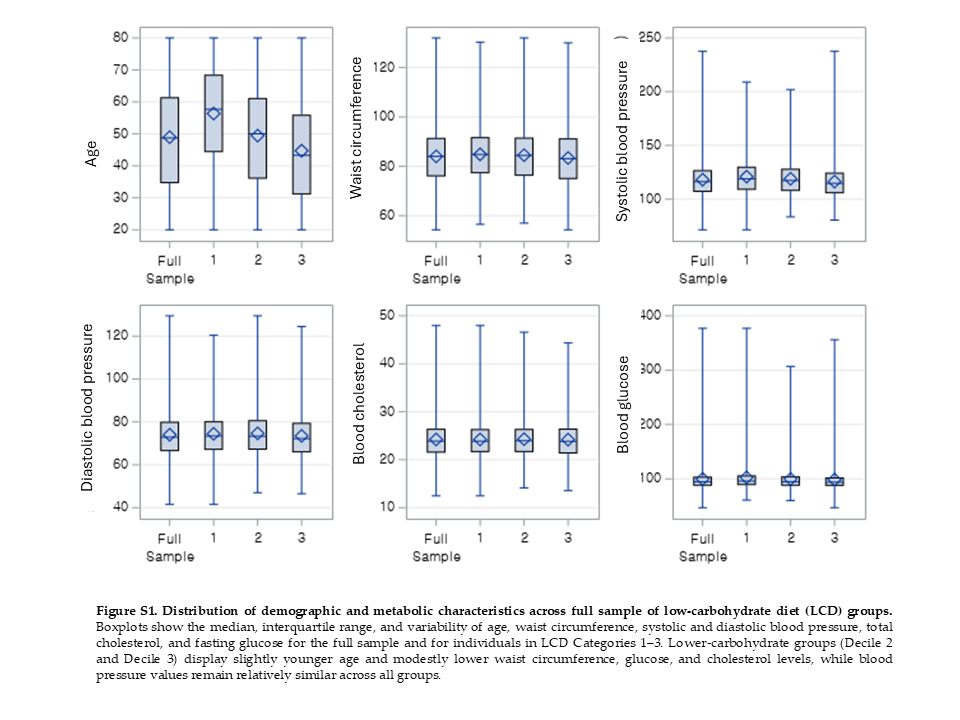

Supplement: Supplementary file 1 [file nutrients-18-00178-s001.zip › nutrients-4058375-supplementary.png]
